# Complete mitochondrial genome of *Rhynchocinetes durbanensis* (Rhynchocinetidae: Rhynchocinetes)

**DOI:** 10.1080/23802359.2016.1157769

**Published:** 2016-03-28

**Authors:** Guopan Tang, Anqun Huang, Gaixiao Qin, Lihui Liu, Guojun Guo, Neng Qin, Zixin Qi, Qiang Lin

**Affiliations:** a College of Animal Science and Technology, Henan University of Animal Husbandry and Economy, Zhengzhou, Henan, PR China;; b Pearl River Fisheries Research Institute, Chinese Academy of Fishery Sciences, Key Laboratory of Fishery Drug Development, Ministry of Agriculture, Key Laboratory of Aquatic Animal Immune Technology, Guangdong Province, Guangzhou, PR China;; c Liuzhou Fishery, Animal Husbandry and Veterinary Bureau, Liuzhou, Guangxi, PR China

**Keywords:** Complete mitochondrial genome, *Rhynchocinetes durbanensis*, shrimp

## Abstract

The complete mitochondrial genome of *Rhynchocinetes durbanensis* (Rhynchocinetidae: Rhynchocinetes) was sequenced in this study. The genome sequence was 17,695 bp in size, with the base composition of 35.14% A, 32.98% T, 20.34% G and 11.55% C of the light strand. The gene order and genes were the same as that found in other previously reported shrimps, including 13 protein-coding genes, 24 transfer RNA genes and two ribosomal RNA genes. Except for *ND5*, *ND4*, *ND4L*, *ND1* genes and eight tRNA genes and two ribosomal RNA genes, all other mitochondrial genes were encoded on the heavy strand. The start codon of *COX1* gene was not determined. These complete mitogenome data provide the basis for taxonomic and conservation research of *Rhynchocinetes durbanensis*, and closely related species.

*Rhynchocinetes durbanensis*, found in the Indo-Pacific, is commonly known as the hingebeak prawn, which belongs to the family *Rhynchocinetidae*. It has many “Y”-shape white spots on the upper front part of its carapace and have features red and white lines on a translucent body (Calado 2009). *Rhynchocinetes durbanensis* was first described scientifically by Isabella Gordon in 1936 (Gordon 1936; De Grave, 2012). *Rhynchocinetes durbanensis* has been frequently confused with *R. uritai* (Okuno & Takeda 1992), and also closely resembles *R. brucei* (Ferrari & Ferrari 2002). As the development of marine aquarium, *R. durbanensis* is popular in the aquarium trade. In this study, we first determined the complete mitochondrial genome of *R. durbanensis*, which contribute to provide basic molecular data to the study on its systematics and conservation biology.

The *R. durbanensis* (RD150801) was collected from Hua Di Wang Flower & Aquarium Market in Guangzhou, Guangdong province in 2015 (23°7'12.00″N 113°15'0.00″E). The specimen stored in Pearl River Fisheries Research Institute, Chinese Academy of Fishery Sciences. Genomic DNA of shrimp tissues was extracted by blood and cell culture DNA kit (QIAGEN, Hilden, FL) and then sequenced using Illumina’s HiSeq 2500 platform (Illumina Inc., San Diego, CA) with 500 bp insert-size DNA library and a pair-end 125 bp sequencing strategy (Miller et al. 2010).

The complete mitochondrial genome of *R. durbanensis* is 17,695 bp in length with an overall base composition of 5835 A (32.98%), 6218 T (35.14%), 2043 G (11.54%) and 3599 C (20.34%) (GenBank accession no. KT590405). The mitochondrial genome of *R. durbanensis* is a circular molecule containing 13 PCGs, 24 tRNA genes and two rRNA genes. 12S-rRNA and 16S-rRNA were located between the *Trna^Leu^* and *ATP8* genes and separated by the *tRNA^Val^* gene, at the length of 1362 bp and 824 bp, respectively. Most of the 39 genes are encoded on the heavy strand (H-strand) except *ND5*, *ND4*, *ND4L*, *ND1* genes, eight tRNA genes and two ribosomal RNA genes, which encoded on the light strand. All tRNA genes can fold into a typical cloverleaf structure, with length ranges from 59 to 70 bp. Five of the 13 protein-coding genes (*COX2*, *ATP6, ND4L, CYTB* and *ATP8*) start with ATG, four of the 13 protein-coding genes (*COX3, ND3, ND5* and *ND2*) start with ATT, *ND6* and *ND1* start with ATA, *ND4* start with GTG, whereas the initiation codon of *COX1* could not been determined. This phenomenon also been found in other shrimps, such as *Rimicaris kairei* (Yang et al. 2012), *Upogebia pusilla* (Shen et al. 2013) and *Farfantepenaeus californiensis* (Shen et al. 2007). All protein-coding genes harboured the typical termination codon TAA.

Some available mitochondrial genome sequences of the congeneric species were used in phylogenetic analysis carried out by maximum-likelihood (ML) method (Figure 1) (Abascal et al. 2005; Posada 2008; Tamura et al. 2011). As expected, *R. durbanensis* was joined with *Neocaridina denticulata* and *Halocaridina rubra* to form a tribe. The reconstructed phylogeny indicated that *R. durbanensis* was closely related to *N. denticulata* and *H. rubra*.

**Figure 1.**
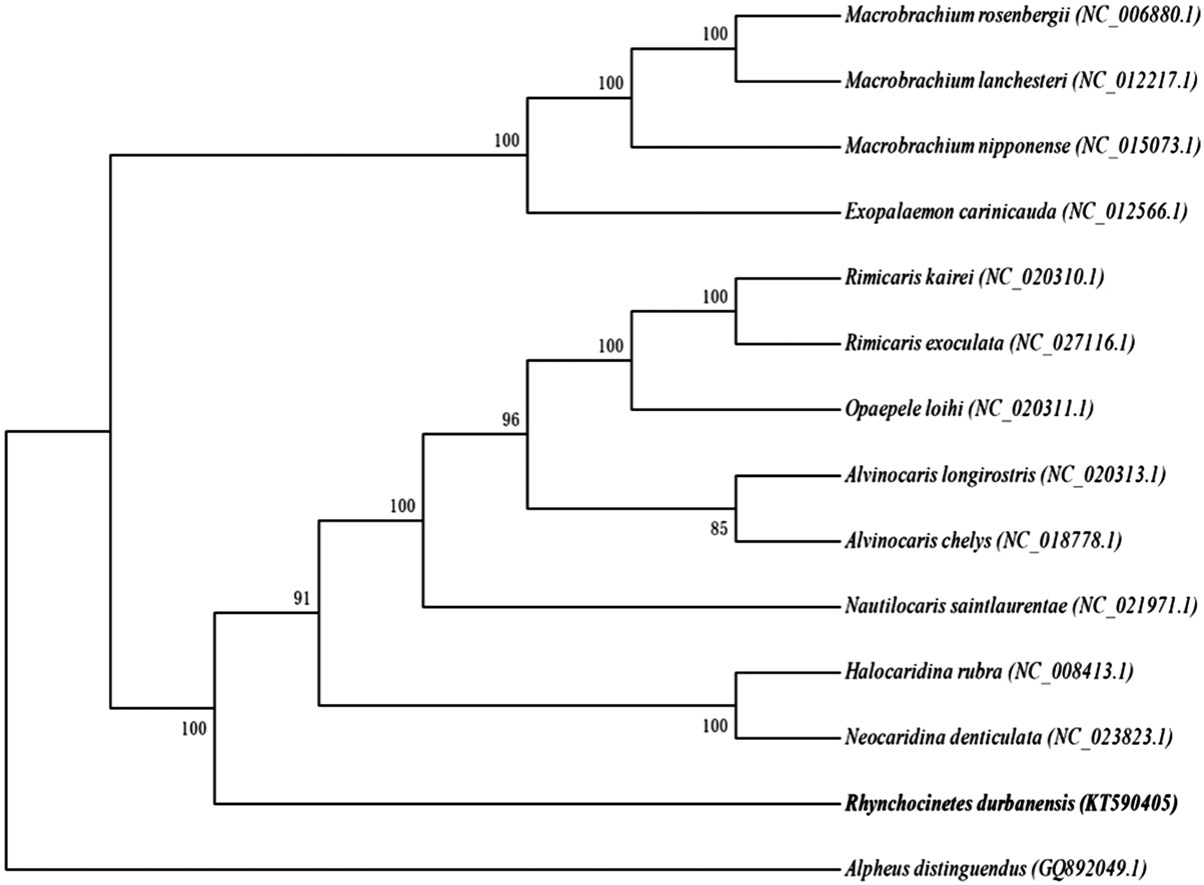
Phylogenetic analysis (ML topology) of *R. durbanensis* based on the whole mitochondrial genome sequences. Numbers at each node represent the bootstrap value for ML analysis.
